# Temporal Stability and Composition of the Ocular Surface Microbiome

**DOI:** 10.1038/s41598-017-10494-9

**Published:** 2017-08-29

**Authors:** Jerome Ozkan, Shaun Nielsen, Cristina Diez-Vives, Minas Coroneo, Torsten Thomas, Mark Willcox

**Affiliations:** 1 School of Optometry and Vision Science, University of New South Wales, Sydney, 2052 Australia; 2 School of Biological, Earth and Environmental Sciences, University of New South Wales, Sydney, 2052 Australia; 3 Centre for Marine Bio-Innovation, University of New South Wales, Sydney, 2052 Australia; 4 Department of Ophthalmology, University of New South Wales, Sydney, Australia

## Abstract

To determine if there is a core ocular surface microbiome and whether there are microbial community changes over time, the conjunctiva of 45 healthy subjects were sampled at three time points over three months and processed using culture-dependent and -independent methods. Contaminant taxa were removed using a linear regression model using taxa abundances in negative controls as predictor of taxa abundances in subject samples. Both cultured cell counts and sequencing indicated low microbial biomass on the ocular surface. No cultured species was found in all subjects at all times or in all subjects at any one time. After removal of contaminant taxa identified in negative controls using a statistical model, the most commonly detected taxon was *Corynebacterium* (11.1%). No taxa were found in all subjects at all times or in all subjects in any one time, but there were 26 taxa present in at least one or more subjects at all times including *Corynebacterium* and *Streptococcus*. The ocular surface contains a low diversity of microorganisms. Using culture dependent and independent methods, the ocular surface does not appear to support a substantial core microbiome. However, consistently present taxa could be observed within individuals suggesting the possibility of individual-specific core microbiomes.

## Introduction

Unlike other regions of the body (gut, skin, mouth), the ocular surface has been thought to be very sparsely and only sporadically colonised by microorganisms due to the potent antimicrobial properties of the tear film^1^ and the relentless mechanical action of the lids^2^. Cultures from swabs of the ocular surface have typically yielded less than 100 bacterial cells^3^. Numerous studies have surveyed the ocular microbiota (lids, conjunctiva and/or tears) and found variations in the types and numbers of organisms that can be cultured from the ocular surface^4–7^. Although normal variations in ocular microbiota exist between individuals and between people from different countries^8^, the microorganisms most commonly cultured include coagulase-negative *Staphylococci* (*S*. *epidermidis* being the most common species), *Proprionibacterium* sp., *Corynebacterium* sp., *Staphylococcus aureus*, and *Micrococcus* sp.^4, 9^. The numbers of cultured organisms recovered is typically lowest from tear film cultures and higher from conjunctival and lid swabs^3^ with pathogenic organisms, such as *Pseudomonas* only sporadically cultured^4, 5^.

Culture-independent methods allow for profiling microbial communities based on differences in the sequence of the 16S ribosomal RNA (rRNA) gene and these have been used to determine the characteristics of the normal ocular microbiota^9–14^. These studies have found a significantly higher microbial diversity than detected by culturing. Some studies noted relatively high abundances of bacteria not previously identified by culturing from normal eyes, including *Rhodococcus erythropolis*, *Erwinia* sp. and *Klebsiella* ^12^. Surprisingly, one of these studies found that many ocular pathogens, including *Pseudomonas* ^13^, which is associated with blinding microbial keratitis^15^, constituted the “core” microbiota of the human conjunctiva. However such conclusion of a “core” microbiota is confounded by the fact that the majority of studies have sampled the ocular surface only at a single time point and those studies, which have sampled longitudinally, investigated only a small subset of healthy subjects, ranging from one^13^, five^12^ to 11^14^ subjects. Another complication, which emerges with 16S rRNA gene sequencing from the low biomass environment of the ocular surface, is the possibility that common contaminants (e.g. from reagents) may be falsely assigned to a “core” of the ocular surface microbiota.

Understanding whether the ocular surface is characterised by a ‘core’ microbiota may shed light on whether idiopathic ocular surface disorders, including dry eye syndrome, Thygeson’s disease, episcleritis, chronic follicular conjunctivitis^16^, all of which have an inflammatory element, could be related to the dysregulation of the ocular microbiome, such as may occur during the short and long-term use of antibiotics. The results of the previous studies left a clear identification of a core ocular microbiome incomplete, as none have examined the stability of the microbiome over time in a large cohort of healthy subjects. In this study, we therefore repeatedly examine the ocular surface in a large cohort of healthy subjects using culture dependent and independent methods. We use presence and relative abundance of taxa within subjects and among times to determine the core microbiome, and further investigate the diversity of the microbiome across individuals, sex and age.

## Results

A total of 45 subjects (22 female, 23 male; mean age 38 ± 10 years) were enrolled into the study, of which 43 subjects were sampled at all three time points (baseline, 1-month, 3-months). Two subjects were permanently discontinued from the study – one due to presumed episcleritis at the 1-month time point and another due to the presence of superficial punctate keratitis, diagnosed prior to sampling at the 3-month time point.

### Culture-based microbial community analysis

**Figure 1:**
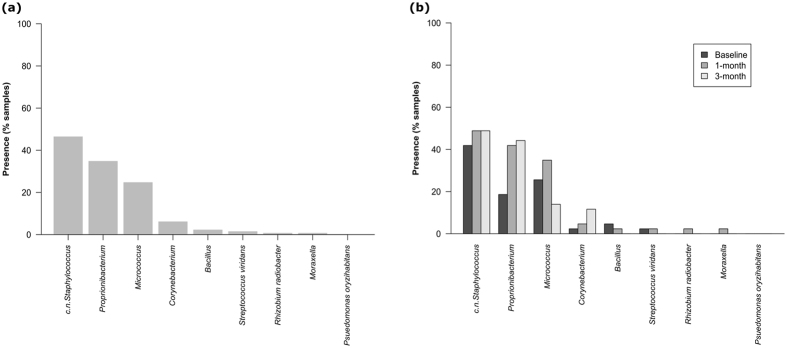
Proportion of samples from which different bacterial taxa were isolated from the ocular surface of subjects sampled at three time points (baseline, 1-month, 3-months), (**a**) in all samples irrespective of time point (n = 129), (**b**) in samples (n = 43) per time point (n = 3).

Using conventional culturing methods, microorganisms were cultured from 76.7% of conjunctival samples across the three-month study period (n = 129). Gram-positive organisms accounted for the majority of isolated microorganisms (94%) with Gram-negative bacteria less frequently isolated (2%). Isolation of fungus occurred sporadically (4%). A total of nine bacterial genera or species were found to be present on the ocular surface. These were from three phyla (*Firmicutes*, *Actinobacteria* and *Proteobacteria*) with over 90% of the isolates belonging to the *Actinobacteria* (52.7%) and *Firmicutes* (41.0%). The most frequently isolated genera from samples were *Staphylococcus* (46.5%), *Proprionibacterium* (34.9%), *Micrococcus* (24.8%) and *Corynebacterium* (6.2%) (Fig. 1a). The most frequently observed bacterial taxa also had the greatest number of colony forming units (CFU) per ocular sample, but at times were also completely absent from a number of samples (Supplementary Fig. 1). A maximum of three species was observed in any one subject. If present, the CFU tended to range between two and 170 per ocular sample with less than 20 CFU found in over 70% of samples.

**Figure 2:**
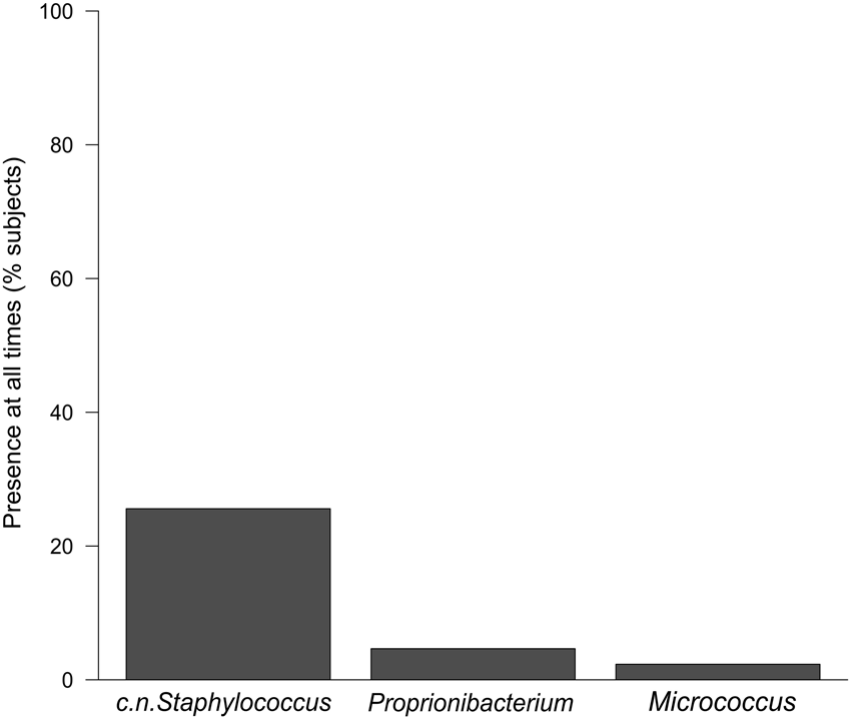
Presence of bacterial taxa in subjects (n = 43) at all three time points.

No species fulfilled the criteria of being present in all subjects at all times (Fig. 1a) or in all subjects in any given time point (Fig. 1b). Coagulase-negative (c.n.) *Staphylococcus*, *Proprionibacterium* and *Micrococcus* were present at all time points in 11 (25.6%), two (4.7%) and one (2.3%) of the subjects, respectively, revealing some longitudinal stability of taxa at an individual level (Fig. 2).

### Culture-independent microbial community analysis

A total of 10,834,099 raw 16S rRNA gene sequences were obtained, which after quality filtering, resulted in a total of 7,395,616 paired end sequences with an average of 54,652 ± 28,912 sequences per sample. These clustered into 62,858 operational taxanomic﻿ unit﻿s﻿﻿ (OTUs) at 97% sequence identity. Following removal of rare OTUs with relative abundances less than 0.0001%, the number was reduced to 11,894 OTUs. Contamination filtering further reduced the number of OTUs to 183 which contained 2,375,176 sequence reads in total (average of 18,556 sequences per sample). Rarefaction curves showed that the majority of samples were sequenced to saturation with a range of 10 to 30 OTUs present per sample (Supplementary Fig. 2).

An average of 16 OTUs were observed on the ocular surface at each sampling time point and there was an average of 33 OTUs per subject across the three time points. We modelled the alpha diversity on the ocular surface against age and sex, and using time points and individuals as random factors. There was significant variation between sample times for the number of OTUs (P = 0.046) (Supplementary Fig. 3). There was however, no significant variation between individuals (P = 0.841), sex (P = 0.220) and age (P = 0.179, Supplementary Fig. 3).

The average Shannon diversity index of the ocular surface at each sampling time point was 1.6. There was significant variation between sampling times for Shannon diversity (P = 0.049) (Supplementary Fig. 4). There was no significant variation between individuals in Shannon diversity (P = 0.100) (Supplementary Fig. 4). There was a difference in the Shannon diversity between sexes, with males having significantly higher Shannon diversity than females (P = 0.040, Supplementary Fig. 4). There was no effect of age on Shannon diversity of the ocular surface (P = 0.929, Supplementary Fig. 4).

**Figure 3:**
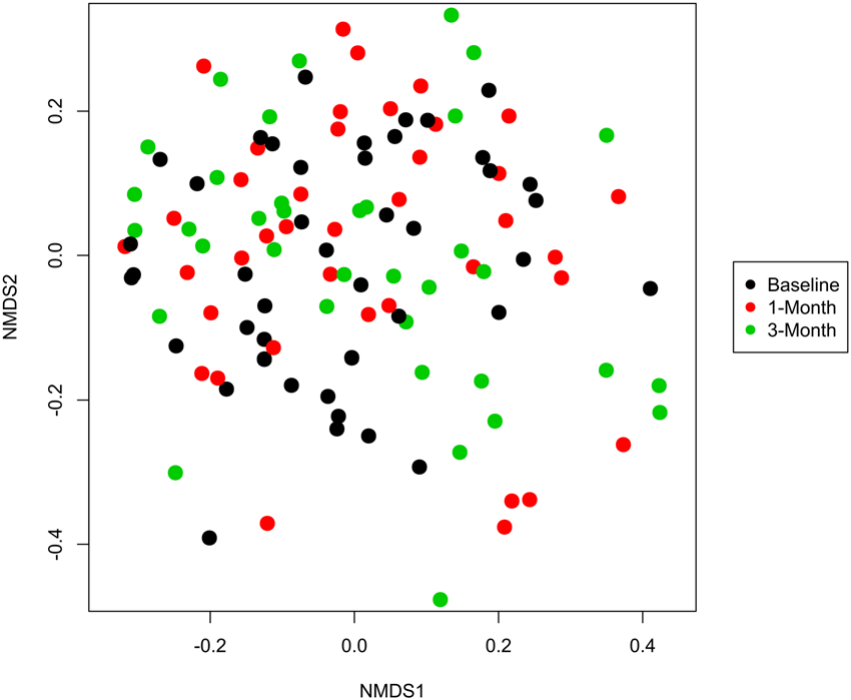
Non-metric multidimensional scaling (nMDS) ordination of the ocular surface microbiome communities by time, compared using Bray-Curtis dissimilarity of OTUs.

Non-metric multidimensional scaling (NMDS) plots using Bray-Curtis dissimilarity of OTUs showed no distinct clustering for bacterial communities of the healthy ocular surface. There was no effect on bacterial community structure for time (Permutational analysis of variance [PERMANOVA] P = 0.878; Fig. 3), age (PERMANOVA P = 0.554) or sex (PERMANOVA P = 0.168). There was however evidence of an effect for individual (PERMANOVA P < 0.001) with significant variation between individual microbiomes (homogeneity of dispersion [PERMDISP] P = 0.033). Testing for interaction between individual and time showed no significant differences (P > 1.00).

### OTU and taxon-based comparison of ocular microbiomes

**Figure 4:**
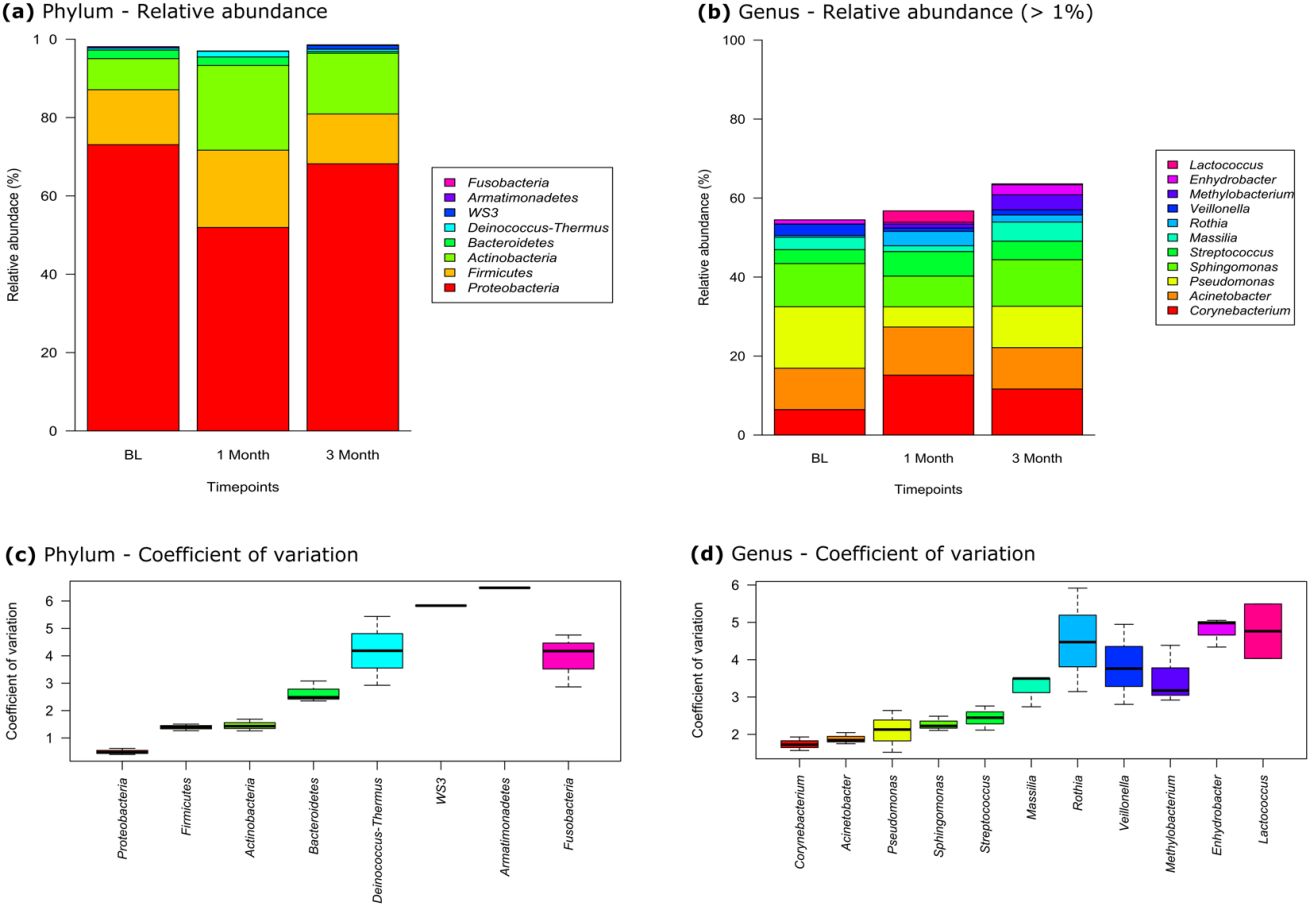
Relative abundance at each time point at the level of, (**a**) phylum, and (**b**) genus (greater than 1% mean relative abundance) and temporal variability in relative abundance in (**c**) phylum, and (**d**) genus (greater than 1% mean relative abundance) using coefficient of variation.

Of the 183 OTUs, 97.8% could be classified to eight phyla, while 73.3% could be classified to 56 genera. Over 90% of OTUs belonged to three phyla: *Proteobacteria* (64.4%), *Firmicutes* (15.5%) and *Actinobacteria* (15.0%) (Supplementary Fig. 5). Approximately 80% of the OTUs belonged to seventeen genera including Corynebacterium (11.1%), *Acinetobacteria* (11.0%), *Pseudomonas* (10.4%), *Sphingomonas* (10.2%), *Streptococcus* (4.8%), *Massilia* (3.2%) and *Rothia* (1.9%) (Supplementary Fig. 5). *Staphylococcus*, which was frequently found with culturing, was less abundant based on the 16S rRNA gene based community analysis, with a relative abundance of 0.2% across all samples. The majority of phyla at each time point consisted of *Proteobacteria* (range 52% to 73%), *Firmicutes* (13% to 20%) and *Actinobacteria* (8% to 22%). The predominant genera at each time point were *Corynbacterium* (range 6% to 15%), *Acinetobacteria* (10% to 12%), *Pseudomonas* (5% to 16%), *Sphingomonas* (8% to 12%) and *Streptococcus* (4% to 6%). The relative abundance of each phylum and genus (Fig. 4a,b) remained relatively stable across the three time points with less variability in relative abundance (using coefficients of variation calculated over time) in the three dominant phyla and the five dominant genera (Fig. 4c,d). There was no significant difference in variance between phyla (P = 0.476) and genera (P = 0.838) across time.

**Figure 5:**
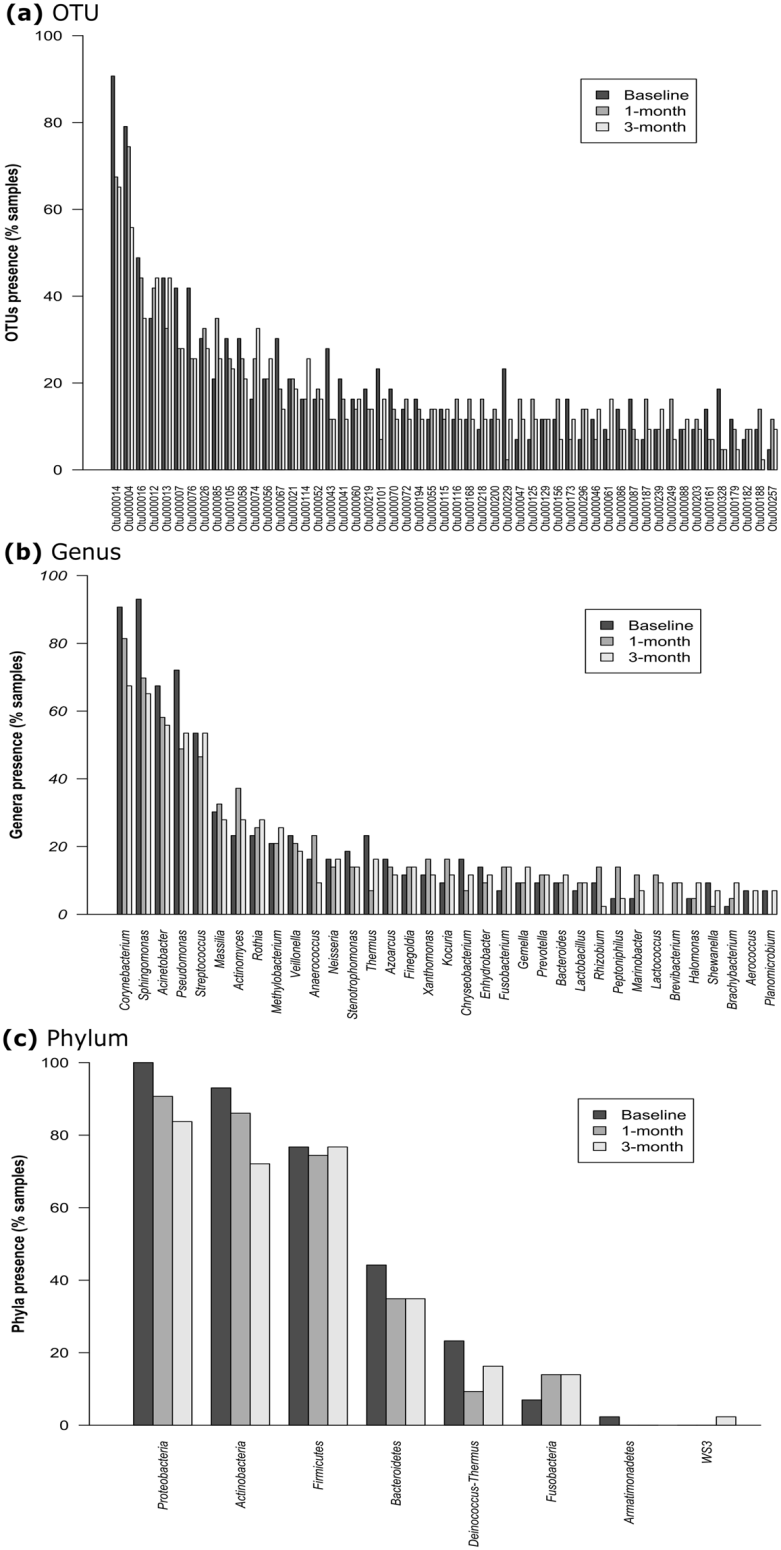
Presence of bacterial taxa in subject samples per time at the level of, (**a**) OTU (data for first 50 shown only), (**b**) genus (first 35 only), (**c**) phylum.

**Figure 6:**
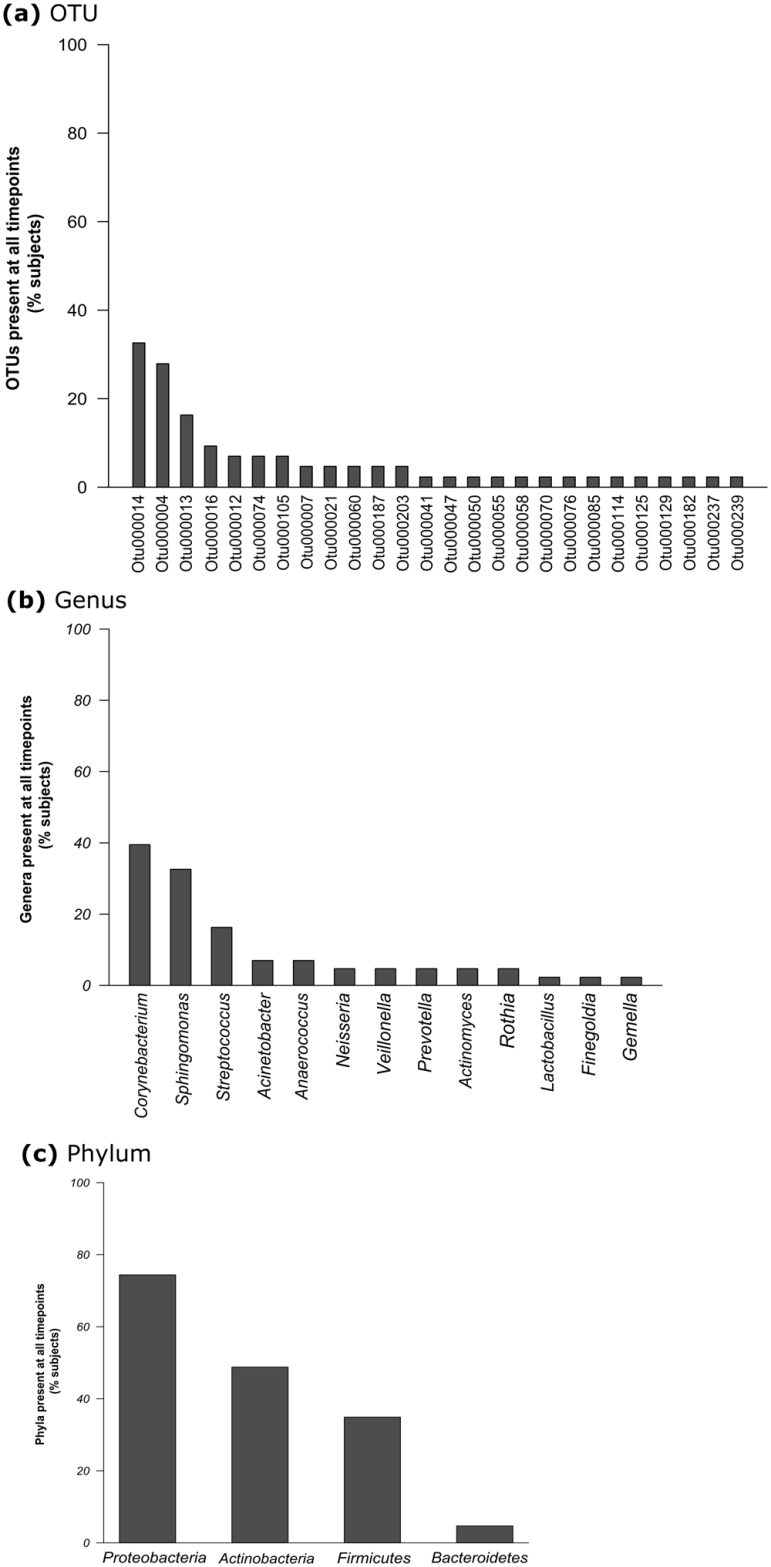
Presence of bacterial taxa in subjects (%) at all three time points at the level of, (**a**) OTU, (**b**) genus, (**c**) phylum.

Irrespective of time point, two OTUs (*Corynebacterium* and *Sphingomonas;* 1.1% of total) were present in greater than 90% of individuals and thirteen OTUs (7.1% of total) were present in 50% of individuals. No OTU fulfilled the criteria of being present in all subjects at all times or in all subjects in any given time point (Fig. 5). There were 73 OTUs present at two out of three sampling periods and 26 OTUs were present in at least one or more subjects at all times (Fig. 6a) and these OTU were assigned to 16 different genera (Fig. 6b) and four phyla (Fig. 6c). The phyla *Proteobacteria*, *Actinobacteria* and *Firmicutes* were present in 74.4%, 48.8% and 34.9% of subjects at all three time points (Fig. 6c). Genera present in subjects at all time points include, *Corynebacterium* (17 subjects [39.5%]), *Sphingomonas* (14 subjects [32.6%]), *Streptococcus* (7 subjects [16.3%]), *Acinetobacter* and *Anaerococcus* (both 3 subjects [7.0%]) (Fig. 6b), indicating some longitudinal stability of taxa at the individual level.

## Discussion

### Towards the true composition of the ocular microbiome

The principal challenge in working with samples containing low microbial biomass is that DNA contaminations can give false-positive signals for the presence of certain microorganism, and in particular if PCR amplification is part of the analysis^17, 18^. Numerous studies have reported contaminating DNA from molecular grade water^19^, PCR reagents^20, 21^ and DNA extraction kits^17, 18, 22^. Contaminant DNA can also come from the laboratory environment and personnel and can be introduced during sampling, DNA extraction, library preparation and/or the sequencing stage. Salter and co-workers found consistent levels of contaminating DNA when sequencing blank controls and with sample dilutions that had been serially diluted to approximately 10^4^ cells^18^. Although contamination may be to some degree unavoidable, the use of suitable controls and careful removal of potential candidate contaminants is therefore necessary when undertaking 16S rRNA gene sequencing of low biomass samples.

How potential contaminations are being dealt with has not been rigorously addressed in the majority of ocular microbiome studies. Zhang *et al*. and Shin *et al*. excluded OTUs using a filtering script in QIIME, which excludes OTU based on low abundance and/or based on OTU presence in the negative control^14, 23^. This is problematic as many of the blank swabs in the current study contained low levels for any given OTU, which would result in discarding the majority of OTUs, including those that are truly present on the ocular surface. Indeed, the most abundant OTUs across our dataset appeared (in low abundance) in the negative controls. Therefore, the contaminant filtering step for the current study removed samples with insufficient sequences (less than 10,000) and removed OTUs present at less than 0.0001% relative abundance. A linear regression analysis used abundances of OTUs in the blank swab and water controls as a predictor of the OTU abundances in the ocular surface samples. Outliers of the regression in greater abundance in individual samples were retained, thus the negative control failed to predict the abundance of retained OTUs as they were most likely truly present in the participant samples and not the negative control. Applying this approach reduced the total number of OTUs identified on the ocular surface from 11,894 to 183. While the majority of 16S rRNA gene based studies have reported higher OTU numbers on the ocular surface, with one study finding an average of 460 OTUs on the healthy conjunctiva and 7,392 OTUs in total from 107 samples^14^, these studies did not apply a rigorous contaminant filtering step. Considering the antimicrobial environment of the ocular surface^1^ and the difficulties involved in generating sufficient amplicon product from ocular surface swabs of healthy individuals compared to individuals with ocular infections^12, 24^, it is unlikely the ocular surface supports such a diverse microbiota as at other body sites. We therefore postulate that the number of OTUs on the ocular surface is in the vicinity of 183, rather than the higher numbers previously claimed, and at most 40 on any given individual.

Using culture methods, the most abundant and prevalent microbial species on the ocular surface were coagulase-negative *Staphylococcus* (phylum *Firmicutes*), *Proprionibacterium* spp., and *Micrococcus* sp. (both phylum *Actinobacteria*), which is in agreement with previous findings^4, 9^. Compared to culturing, significantly greater bacterial diversity was found with high-throughput sequencing. In our case, 183 OTUs which covered 8 phyla and 56 genera (with 17 genera possessing a relative abundance greater than 1%) which still indicates a substantial microbial diversity, in contrast to culture-dependent study that were only findings 9 genera. The caveat here is that culture-independent methods do not distinguish between viable and non-viable microorganisms in contrast to culture methods. Furthermore, not all viable microorganisms can be cultured on the two types of growth media used.

### Factors that influence microbial composition of the ocular microbiome

Overall ocular surface microbial alpha diversity measures of richness and Shannon diversity were low and relatively stable over time. Another study which assessed the eyelid margin and tears of individuals at a single time point also found low variability within individuals^11^. Such constant low alpha diversity may be due to the constant purging of the ocular surface by the mechanical action of the lids. A statistically significant increase in microbial richness and Shannon diversity index was observed over time, which was possibly related to the variability inherent in randomly sampling only a limited area of a low biomass surface and the sparse, transient and regional ocular surface microbiome that is exposed to the external environment and constantly interacting with the lid margin/skin microbiota. Another potential cause of temporal variability could be due to differences in eye sampling allocation at each time point and/or possible microbial population differences between the two eyes. Although no culture independent study has compared the ocular microbiota between the two eyes, a culture based study found no significant difference in conjunctival biota between eyes using normal culturing techniques^25^.

There was no effect of age on richness (number of taxa) and Shannon diversity index (number of taxa and evenness of their distribution) over time. Although there was no difference in richness for sex, there was, albeit small, a difference in Shannon diversity over time with males having a higher index compared to females. A study by Zhou *et al*. found no effect of sex on richness and Shannon index, but found significantly higher richness and Shannon index in children aged less than 10 years compared to an older age group^26^. Zhou *et al*. however sampled individuals at a single time point and the age range was significantly younger (age stratified below and above 10 years of age) compared to the present study (age range 22 to 64). Zhou *et al*. suggested that the close physical contact among younger children, reduced immunity and hygiene differences (compared to adults) may be factors in the increased microbial diversity observed for this age group^26^.

Compared to the relative stability in alpha diversity within individuals, there was a significant difference in beta diversity between individuals. Numerous ocular microbiome studies have also reported higher variability in microbial diversity between individuals compared to within individuals^11, 13, 14^. Assessment of the bacterial composition across other body habitats including the gut, oral cavity, nostrils, external auditory canal, head hair and various skin surface locations^27^ have also found microbial communities to be more stable within individuals and highly variable among individuals^27^. There was no difference in microbial community structure with repeated sampling of the healthy ocular surface by time, sex or age. A recent study by Shin *et al*., reported no differences in ocular surface bacterial diversity over time or individual in a subgroup (n = 11) of healthy non-contact lens wearing subjects sampled biweekly over 6 weeks^14^. However, Shin *et al*. did report an effect of sex on the ocular surface microbial community structure^14^.

### Is there a core ocular microbiome?

There was an average of 16 OTUs at each sampling with an average of 33 OTUs per individual in total across the three sampling time points. No OTU however was identified to be present in all individuals at all time points or in all individuals in any one time point. This suggests that unlike other mucosal surfaces on the human body, such as the skin^28^, gut^29^, vagina^30^ or oral cavity^31^, which possess a signature core microbiome of OTUs, the ocular surface is not colonised consistently by any given OTU. Although there was greater transience at the OTU and genera level, greater commonality was observed at the phylum level with most OTUs (94.9%) found on the ocular surface related to three phyla: *Proteobacteria* (64.4%), *Firmicutes* (15.5%) and *Actinobacteria* (15.0%). Dong *et al*. also found these three bacterial phyla to have the highest relative abundance on the conjunctiva^13^. OTUs associated with the genera *Corynebacterium* (phylum *Actinobacteria*), *Streptococcus*, *Anaerococcus* (both *Firmicutes*), *Sphingomonas* and *Acinetobacter* (both *Proteobacteria*) were found on one or more individuals at all time points. Overall, OTUs belonging to the genera *Corynebacterium* were the most prevalent, being found in approximately 40% of individuals at all time points and 90% of individuals irrespective of time point. Recent ocular microbiome studies of healthy eyes have found OTUs associated with *Corynebacterium* to be the most abundant on the ocular surface^9, 14, 26^. Similarly, an analysis by Doan *et al*., found *Corynebacterium* OTUs to have the highest statistical likelihood of originating from the conjunctiva and not derived from the environment. *Sphingomonas*, *Streptococcus*, *Pseudomonas and Acinetobacter* have all previously been reported as being part of the ocular microbiota^9, 13, 14, 26^. The origin of *Corynebacterium* is likely the skin/lid margins and nasal cavity, where it is dominant^28, 32^, while *Streptococcus* is commensal to the oral cavity^33^. The origins of *Sphingomonas*, *Acinetobacter* and *Pseudomonas* are thought to be external to the body. Interestingly, some species of these microorganisms are highly resilient to a range of antimicrobials^34, 35^, which may perhaps give them an advantage over other bacteria on the hostile surface of the eye. Recent animal studies have shown that commensal microorganisms (including *Corynebacterium* and *Streptococcus*) can exist in the ocular mucosa and appear to be immunologically relevant with the function of promoting immunity to pathogens (*P*. *aeruginosa*, *C*. *albicans*) by facilitating recruitment of neutrophils^36, 37^. Upon infection, corneas with commensals had less severe pathology scores compared to corneas with reduced commensals (induced with antibiotic treatment) suggesting commensals contributed to infection resistance^38^.

The species most commonly cultured from the ocular surface was coagulase-negative *Staphylococcus* spp. This taxa was also detected by 16S rRNA gene sequencing, but at low relative abundances (0.2%), which indicates that culturing possibly overestimates the true abundance of *Staphylococcus* spp. Previous sequencing studies of the healthy conjunctiva have also reported a low relative abundance (approximately 2%) of *Staphylococcus* on the ocular surface^13, 26^, while other studies have found *Staphylococcus* to be more abundant (13.2%)^9^. One study reported the presence of *Klebsiella* (phylum *Proteobacteria*) with repeat sampling of five healthy individuals over a 3-month period using 16S rRNA sequencing and suggested that it may be part of the normal ocular flora^12^. In contrast, only a single sequence of *Klebsiella* was identified (prior to contaminant filtering) in one individual at one time point in the current study. Following contaminant filtering, *Klebsiella* was not present and hence was not considered part of the ocular microbiome.

Understanding the characteristics and temporal variation of the normal ocular surface microbiome is important for establishing baselines from which differences caused by disease, antibiotic use or contact lens wear can be detected. Changes in the ‘core’ microbiome could be correlated to disease states or increased risk to ocular health^16^. To establish such baseline data, this is the first study to assess the temporal stability of the ocular surface microbiota using both standard culturing and 16S rRNA gene sequencing on a relatively large cohort of healthy, non-contact lens wearing subjects without the use of a topical anaesthetic, which has been shown to reduce alpha diversity and alter microbial community composition and structure^14^. Hamady and Knight have defined different models of a core microbiome, ranging from a “substantial core” (where most individuals share most of the taxa) and “minimal core” (all individuals share a few taxa) to a “no core” model (no taxa shared across individuals)^39^. Unlike other surfaces of the human body, the results of the current study suggest there is a low diversity of microorganisms on the ocular surface which does not appear to support a “substantial” core microbiome. Using a statistical model to differentiate contaminant taxa from ocular surface taxa, the consistent presence of taxa within individuals over time suggests the possibility of individual-specific (or “minimal”) core microbiomes^39^. How these microbial populations reach the ocular surface, their function and viability whilst on the surface and whether they are found on the ocular surface over even longer periods of time should be the subject of future studies.

## Methods

### Inclusion/Exclusion Criteria and Visit Schedule

The study protocol was reviewed and approved by the Human Research Ethics Committee of The University of New South Wales and the research followed the tenets of the Declaration of Helsinki. Informed consent was obtained prior to enrolment of subjects into the study. Subjects were required to be over 18 years of age, not wear contact lenses 3 months prior to and during the study period, have no ocular or systemic disease, no history of eye trauma or surgery (including corneal refractive surgery), no use of antibiotic, anti-inflammatory or immunosuppressive medication in the past 6 months. To screen for dry eye, all subjects were required to complete the Ocular Surface Disease Index Questionnaire^40^ at the baseline visit. Only subjects with a score below (and including) 13 were permitted to continue in the study.

### Sample Collection

Previous ocular microbiome research has utilised mattress design swabs (of either cotton or rayon material), but these have been shown to have worse outcome in terms of retrieving and releasing cellular material^41, 42^. This may not be so significant when sampling sites of high microbial loads, but is particularly critical for sampling the low microbial load of the ocular surface, with one recent study confirming that use of nylon flocked swabs significantly improved DNA recovery from samples with low quantity using a manual DNA extraction method^43^. A series of pilot studies utilising swabs inoculated with serial dilutions of *Staphylococcus epidermidis* showed improved yield with flocked swabs compared to mattress swabs for recovery of low levels of microbial DNA (data not shown). Furthermore, vortexing of mattress swabs may not be effective at fully releasing cellular material (Zain 1995) and use of the Human Microbiome Protocol, where the swab is vortexed then rolled around the inside of the collection tube prior to removal, is also likely to significantly reduce biomass yields^44^. The use of a flocked swab and centrifugation of the swab contents into the lysis solution (while the swab head and shaft remained above the solution) maximised the release of its biomass content (details in *DNA extraction* section below).

The health of the ocular surface and eyelid margin was assessed for subjects prior to sampling. Each eye was sampled with a single, sterile, nylon, flocked swab (FLOQSwabs; Copan, Brescia, Italy) and all sampling was conducted by the same clinician (JO) to ensure consistency. Each swab was wiped first across the upper then the lower bulbar conjunctiva, three times each, avoiding any contact with the lid margin. The swab was rotated in the direction opposite to the direction of the wipe to maximise collection efficacy. The swab from each eye was randomly assigned to be processed for either microbial culturing or DNA analysis at the baseline, 1 and 3 month visits. Swabs assigned to microbial culturing were placed in vials containing 2 ml phosphate buffered saline and were processed within 1 hour. Swabs allocated for 16S rRNA gene analysis were placed in a microcentrifuge tube and immediately frozen using a −20 °C LabTop Cooler (Thermo Fisher Scientific, Coralville, MA, USA) and then transferred to a −80 °C freezer within 1 hour and processed within 6 months of collection. A total of nine negative control swabs (swabs removed from packaging in proximity to the subject and placed directly in a microcentrifuge tube) were collected during the study period.

### Microbial Culturing

The vial containing the swap sample was vortexed for 30 seconds at high speed. After aseptic removal of the swab, 400 µl aliquots were inoculated onto three chocolate blood agar (CBA) (Oxoid, Basingstoke, United Kingdom) plates and one Sabouraud dextrose agar (SAB) plate (Oxoid). The three CBA plates were incubated at 37 °C aerobically for 48 hours, in 5% CO_2_ for 48 hours or under anaerobic conditions for 96 hours. The SAB plates were incubated at ambient conditions (22 °C) for 7 days to culture fungi. Each macroscopically different microbial colony was then subjected to standard microbial identification procedures including Gram staining, standard biochemical methods and API kits (bioMerieux, Marcy-l'Étoile, France) for Gram-negative isolates^45^.

### DNA Extraction

Genomic DNA was extracted using the NucleoSpin Tissue XS kit (Machery-Nagel, Düren, Germany) with the following modifications. The microcentrifuge tube containing the swab was removed from the freezer and 76.8 µl of lysis buffer (T1) was added to the tube and vortexed for 30 seconds and then centrifuged for 30 seconds at 17,000 × g. Using aseptic techniques, the tip of the swab shaft was gripped with a tweezer, bent over the lip of the microcentrifuge tube (keeping the tip of the swab head above the 100 µl marker level) and the tube lid tightly closed over the swab shaft. The tube was then centrifuged at 17,000 × g for 2 minutes, after which the swab was discarded. This ensured that all potential microorganisms or lysed cell material on or in the swab were expelled into the lysis solution. To ensure complete lysis, the manufacturer’s protocol was modified with the addition of 3.2 µl of freshly prepared lysozyme (10 mg/ml) (Sigma-Aldrich, Merck KGaA, Darmstadt, Germany) into the lysis buffer. The solution was briefly vortexed then incubated at 37 °C for 30 minutes. Then 8 µl of Proteinase K (20 mg/ml) was added to the solution, vortexed, incubated at 56 °C for 1 hour and vortexed again. The manufacturer’s protocol was then followed.

Amplification of the V4 region of the 16S rRNA gene was performed using primers 515 F (5′-GTG BCA GCM GCC GCG GTA A-3′) and 806R (5′-GGA CTA CHV GGG TAT CTA ATC C-3′). The final PCR reaction volume was 25 µl consisting of 0.5 µl each of forward and reverse primers (Integrated DNA Technologies, Coralville, IA, USA), 7.5 µl of nuclease free water (Thermo Fisher Scientific), 12.5 µl EconoTaq Plus Green 2x MasterMix (Lucigen,Middleton, WI, USA) and 4 µl of DNA extracts. A touchdown PCR protocol was used to increase primer annealing specificity and yield^46^. The touchdown phase consisted of an initial denaturation step at 94 °C for 3 minutes, denaturation at 94 °C for 20 seconds, annealing at 69 °C for 30 seconds and extension at 72 °C for 40 seconds for 12 cycles with the annealing temperature decreased by 1 °C per cycle. At the conclusion of the touchdown phase, the protocol involved denaturation at 94 °C for 20 seconds, annealing at 58 °C for 30 seconds and extension at 72 °C for 40 seconds. This was repeated for 23 cycles and followed by a final extension at 72 °C for 5 minutes. Positive (*E*. *coli* genomic DNA) and negative (nuclease free water and blank swabs) controls were included. The PCR products were assessed by electrophoresis on a 1% agarose gel using 8 µl of the amplified product. A 1kB DNA ladder (Gene Ruler, Thermo Scientific) was used to confirm the size and approximate the amount of the PCR product. Gels were imaged with a Gel Doc XR+ (Bio Rad, Hercules, CA, USA) and the luminosity of the PCR bands was determined using Windows Paint software (Microsoft, Seattle, WA, USA).

The 16S rRNA gene PCR amplicons were purified and libraries were prepared using Illumina sequencing adaptors and dual indices. Paired-end amplicon sequencing (2 × 250 bp) was performed on the Illumina MiSeq platform at the Ramaciotti Centre for Genomics (University of New South Wales, Sydney, Australia).

### Sequence quality and abundance filtering

The 16S rRNA gene sequencing quality scores were examined with FastQC (www.bioinformatics.babraham.ac.uk/projects/fastqc/). The sequences were analysed and quality filtered using Mothur version 1.37.1^47^ following the Mothur MiSeq standard operating procedure^48^. Briefly, paired-end sequences were formed into contigs, aligned against the SSU SILVA database version 123 (www.arb-silva.de)^49^, removed of chimeric sequences using UCHIME^50^, and taxonomically classified using the Ribosomal Database Project taxonomic outline^51, 52^. Sequences classified chloroplast, mitochondria, eukaryote and unknown were removed, as too were singleton reads at this stage of the quality filtering pipeline. Sequences were clustered in operational taxonomic units (OTUs) at 97% similarity, and a consensus taxonomy was obtained from the classification of sequences within an OTU.

### Contaminant filtering

The low biomass of microbial material from the ocular surface﻿ samples﻿﻿﻿ posed a risk that material from the environment and reagents could contaminate the analysis of the microbial composition of the ocular surface. Potential contaminating sequences were therefore removed with the following steps: (a) samples with insufficient sequences (less than 10,000) were removed (Supplementary Fig. 6); (b) OTU abundances were standardised by converting sequence counts to relative abundances within each sample; (c) the relative abundance was multiplied by the relative PCR band luminosity (as a surrogate measure of target DNA concentration and hence target microbial biomass from the sample). The relative PCR band luminosity was determined for each sample by taking the difference in the luminosity of each PCR band from the band with the highest luminosity and then dividing this value by the highest luminosity value; (d) the mean relative abundances across the three sample types (subject samples, blank swab control and nuclease-free water control) were calculated; (e) OTUs present at less than 0.0001% of the total relative abundance were removed (Supplementary Fig. 7); (f) linear regression analysis was then conducted using the relative abundances of all OTUs in the blank swab negative control as explanatory variable and the relative abundance of the same OTUs in the subject samples as dependent variable (relative abundances in the subject samples); (g) the prediction standard error (SE) from the linear regression were used as a filter threshold, with the SE multiplied by 5 and the exponent calculated. OTU outliers of the regression that were in greater abundance in subjects samples were retained for further filtering (Supplementary Fig. 8a); (h) the linear regression filter was repeated using the retained OTUs and the outliers of the regression that were in greater abundance in the subjects samples were retained (Supplementary Fig. 8b); (i) the linear regression filter was applied on the retained OTUs using the OTUs obtained from the nuclease-free water samples as the predictor following the steps outlined in f-g. (Supplementary Fig. 9). The final retained OTUs were considered to be truly present in the participant samples and used in downstream statistical analysis.

### Core microbiome

The presence/absence of taxa were compared among and within subjects, and across sampling times. Core microbiome was defined as being present in all subjects at all time points.

### Community analysis

Rarefaction analysis was used to determine if a complete representation of the ocular surface microbiome had been achieved given the observed sequence sampling depths. Levene’s test was used to assess homogeneity of variance between phyla and genera relative abundance across time. Microbial alpha- and beta- diversities were assessed using methods within the Vegan R package for community ecology analysis^53^. The microbial species diversity (alpha diversity) was described by the metrics of richness (the number of OTUs) and Shannon index (a measure of diversity which takes into account richness and relative abundance of each taxa, and where a higher index indicates an increased number of OTUs with higher evenness between OTUs). The microbial beta-diversity was compared using the Bray-Curtis dissimilarity coefficient. The microbial alpha diversity metrics were compared using normal linear mixed models (R package lme4) with age and sex as fixed effects and time and subject as random effects and ANOVA to test for significance. A normal (Gaussian) linear mixed models was fitted and the model fits were checked using residual plots. The scatter of residuals showed no pattern, suggesting that the data fit the model and hence conclusions drawn from the models were valid. Microbial beta diversity was compared across time, subject, age and sex using PERMANOVA. Statistical analysis of culture dependent and independent results was performed using R statistical software, version 3.1.3 (http://cran.r-project.org/).

## Electronic supplementary material

Supplementary Information
