# Supplementary material for: Temporal Stability and Composition of the Ocular Surface Microbiome
Source: Sci Rep. 2017 Aug 29;7:9880. doi: 10.1038/s41598-017-10494-9 (PMC5575025; doi:10.1038/s41598-017-10494-9)

Supplementary Information

Temporal Stability and Composition of the Ocular Surface Microbiome

Jerome Ozkan, Shaun Nielsen, Cristina Diez-Vives, Minas Coroneo, Torsten Thomas, Mark Willcox

Supplementary Figure 1: Colony forming units per swab of bacterial taxa isolated from the ocular surface from 43 subjects sampled monthly for three months (n = 132)

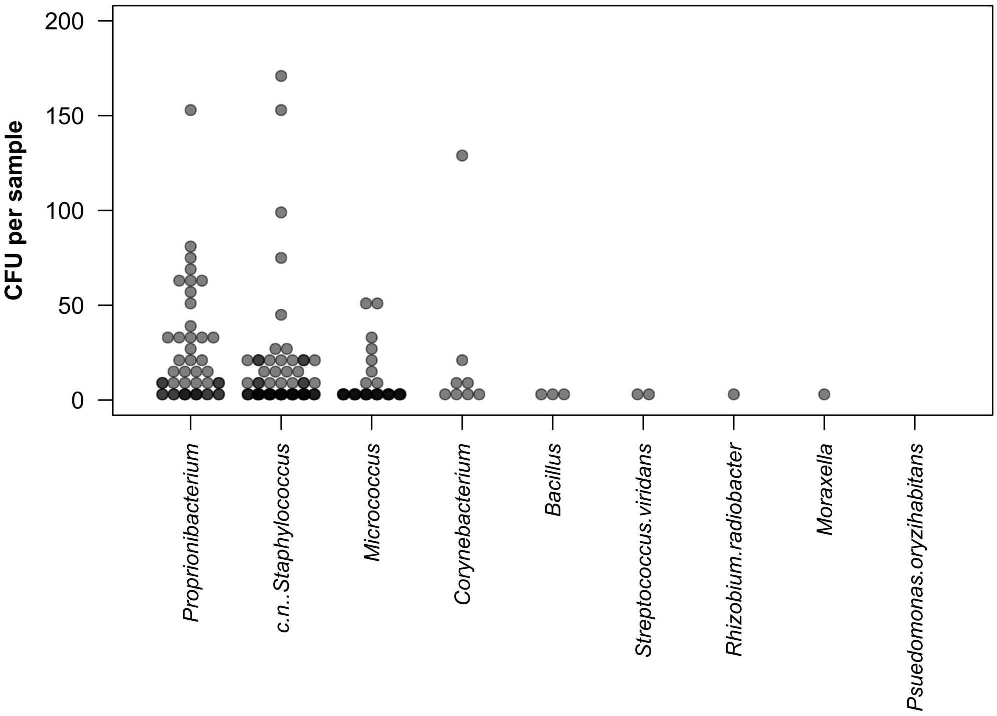

Supplementary Figure 2: OTU rarefaction

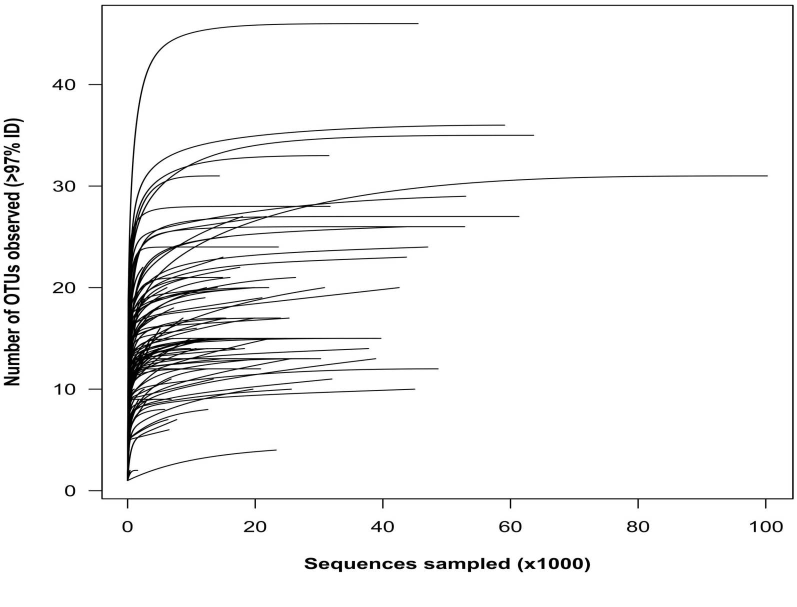

Supplementary Figure 3: Microbial richness at OTU level for **(a)** time, **(b)** individuals, **(c)** sex, and **(d)** age

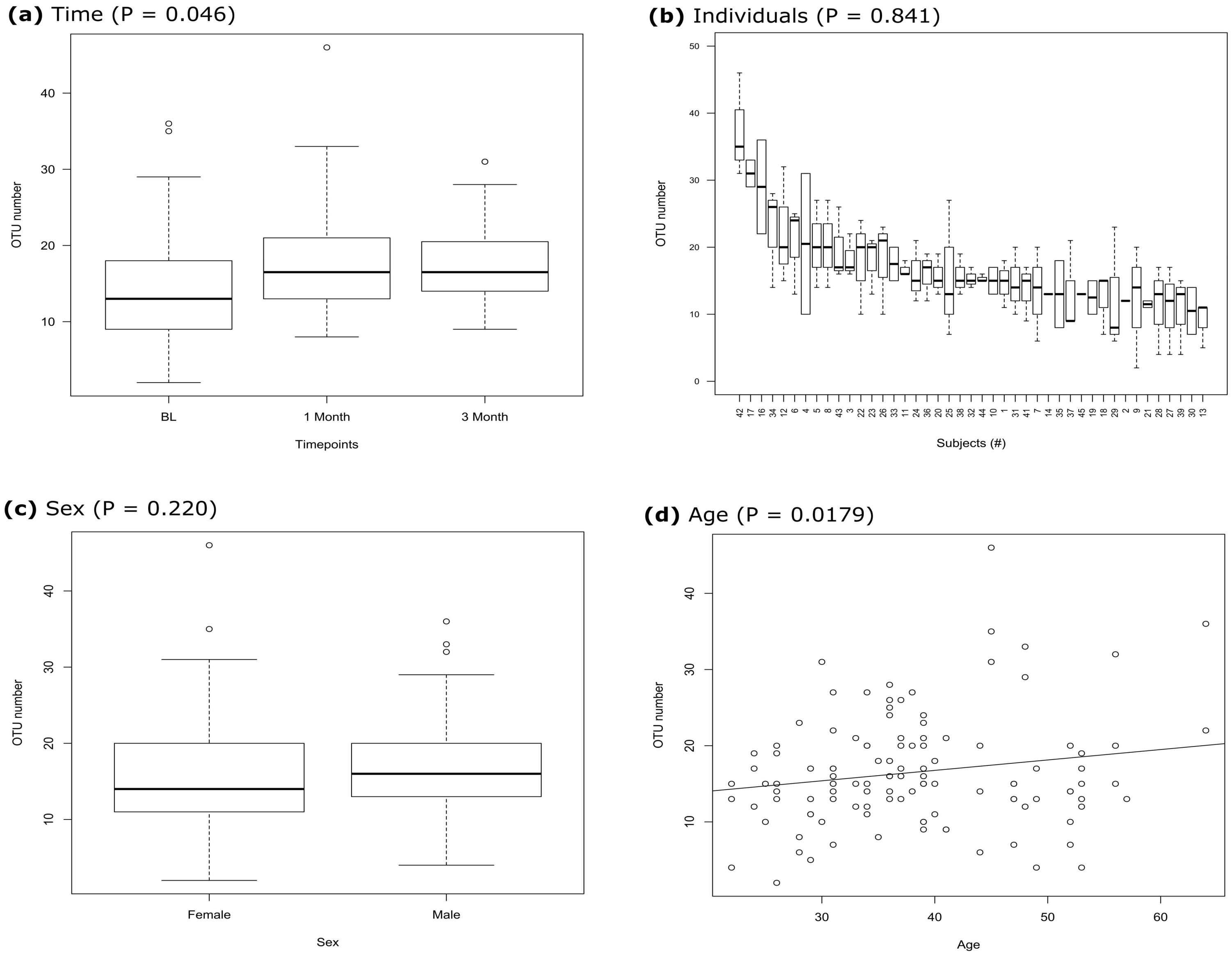

Supplementary Figure 4: Shannon diversity index at OTU level for, **(a)** time, **(b)** individuals, **(c)** sex and **(d)** age

(a) Time (P = 0.049)

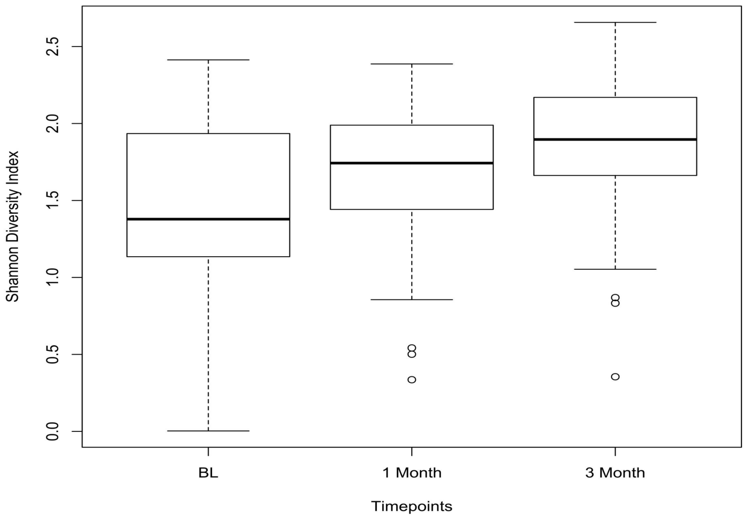

(b) Individuals (P = 1.000)

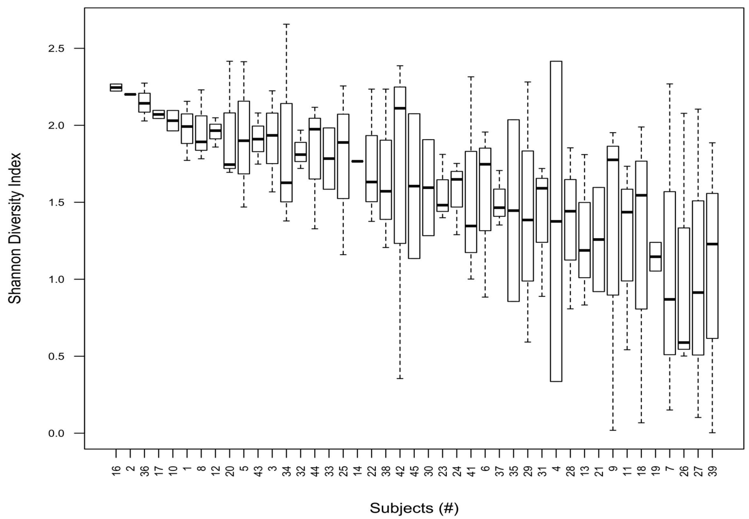

(c) Sex (P = 0.040)

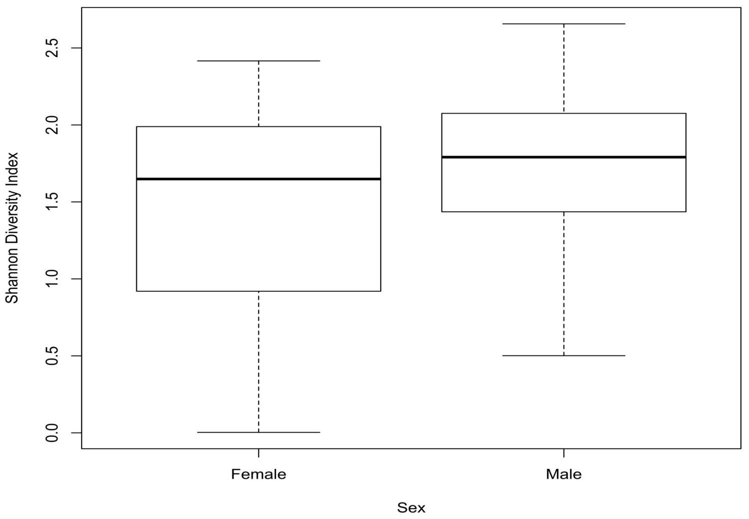

(d) Age (P = 0.929)

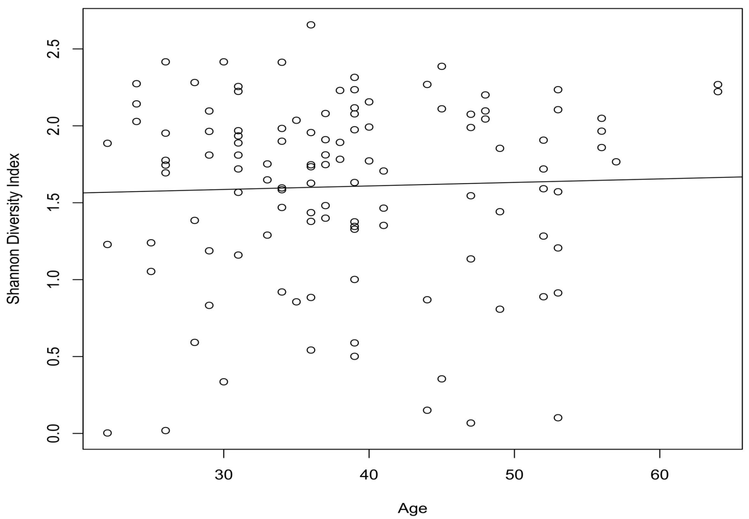

Supplementary Figure 5: Relative abundance at the level of, **(a)** phylum**, (b)** genus

(a) Phylum

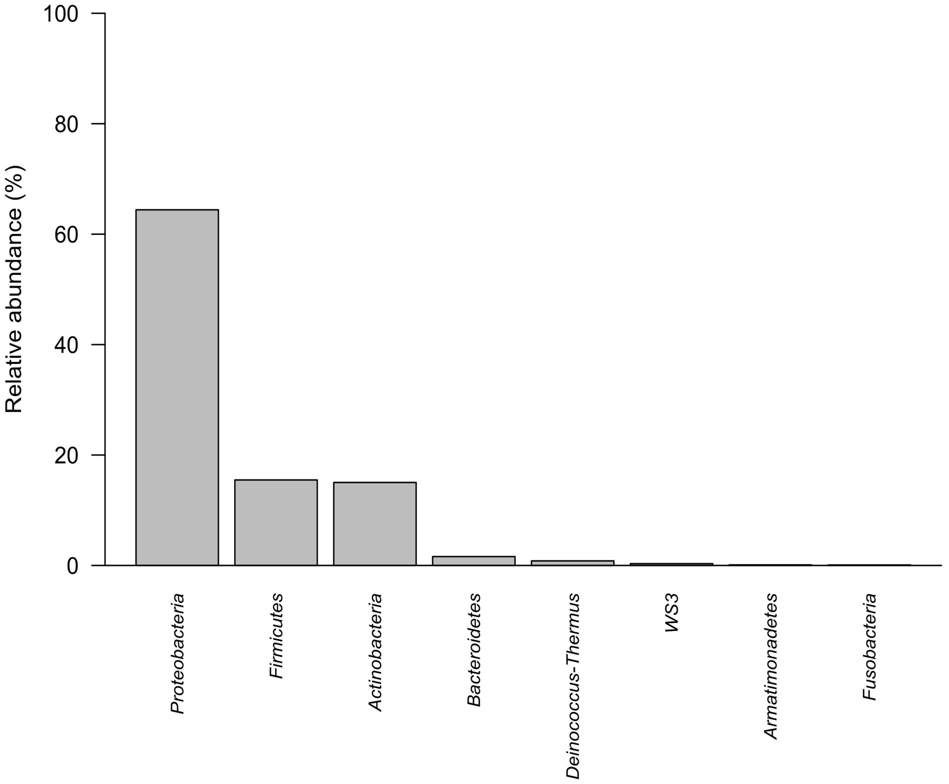

(b) Genus

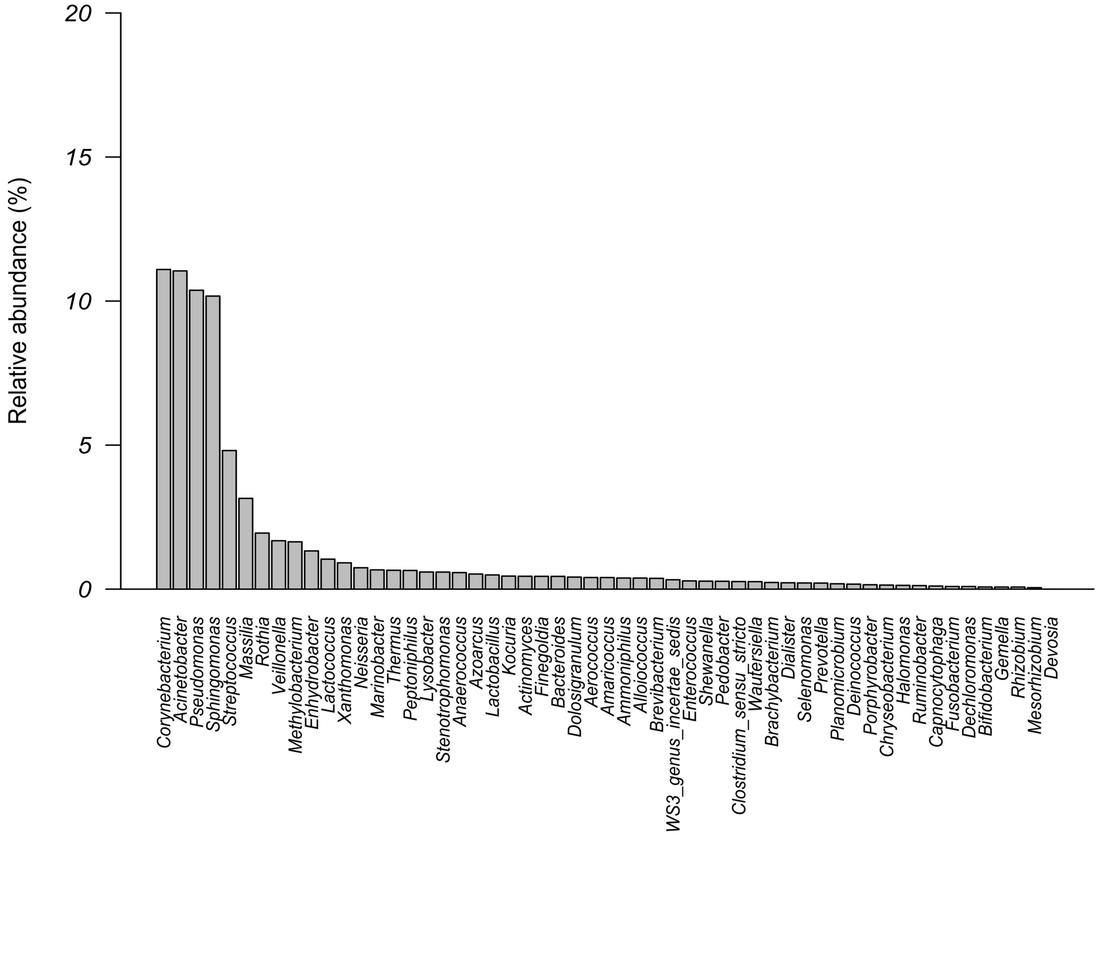

Supplementary Figure 6: Sequence counts (Log10) per sample. Samples with < 10,000 sequences (indicated by dashed line) were removed.

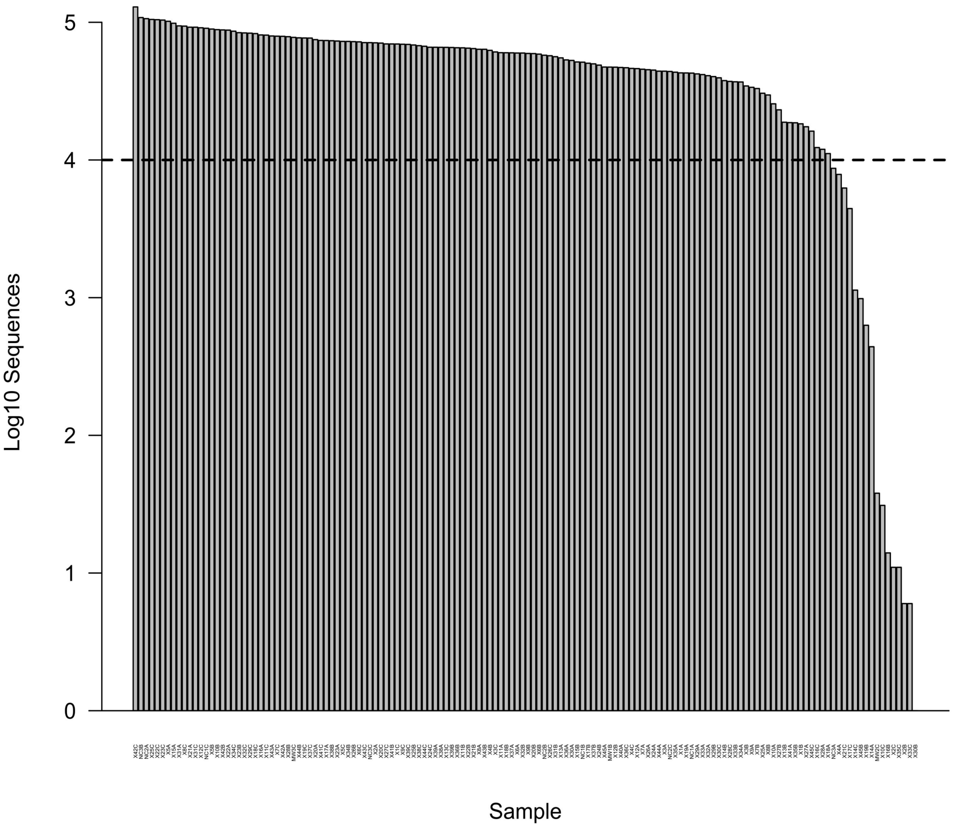

Supplementary Figure 7: OTU relative abundance. OTUs present at less than 0.0001% relative abundance (indicated by dashed line) were removed.

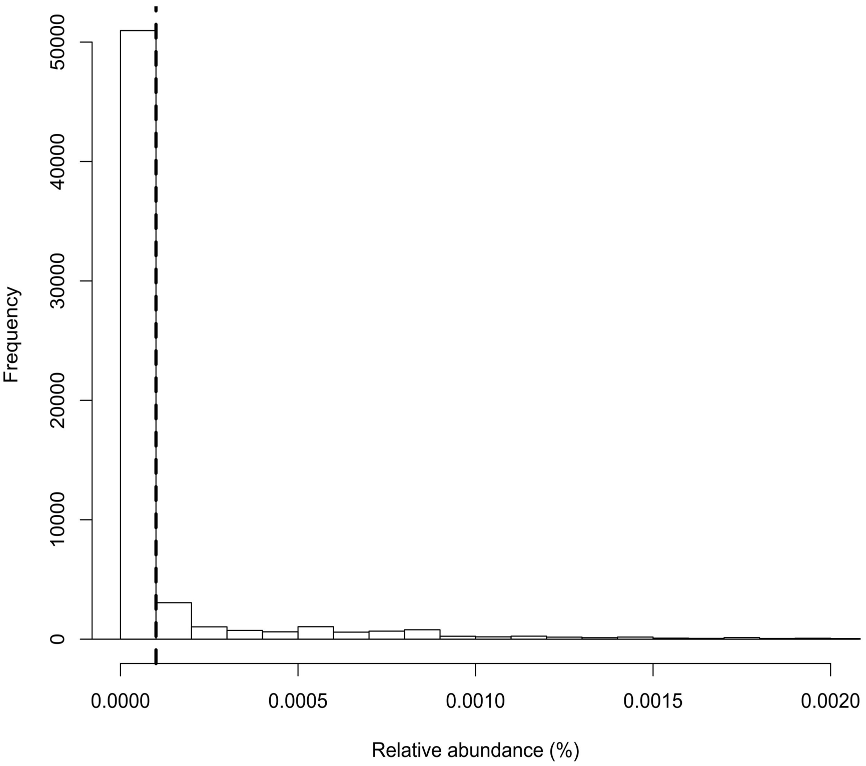

Supplementary Figure 8: Linear regression filter to determine OTUs present on ocular surface samples, **(a)** Initial linear regression filter, **(b)** Linear regression filter repeated (OS = ocular samples, NC = negative control samples, Black = 1:1 line, Red = regression line, Green = standard error [SE] line, Blue = SE multiplied by a factor of five, Aqua = exponent of 5x SE; Black circles = OTUs; Green points = retained OTUs after initial filter; Aqua points = final retained OS OTUs)

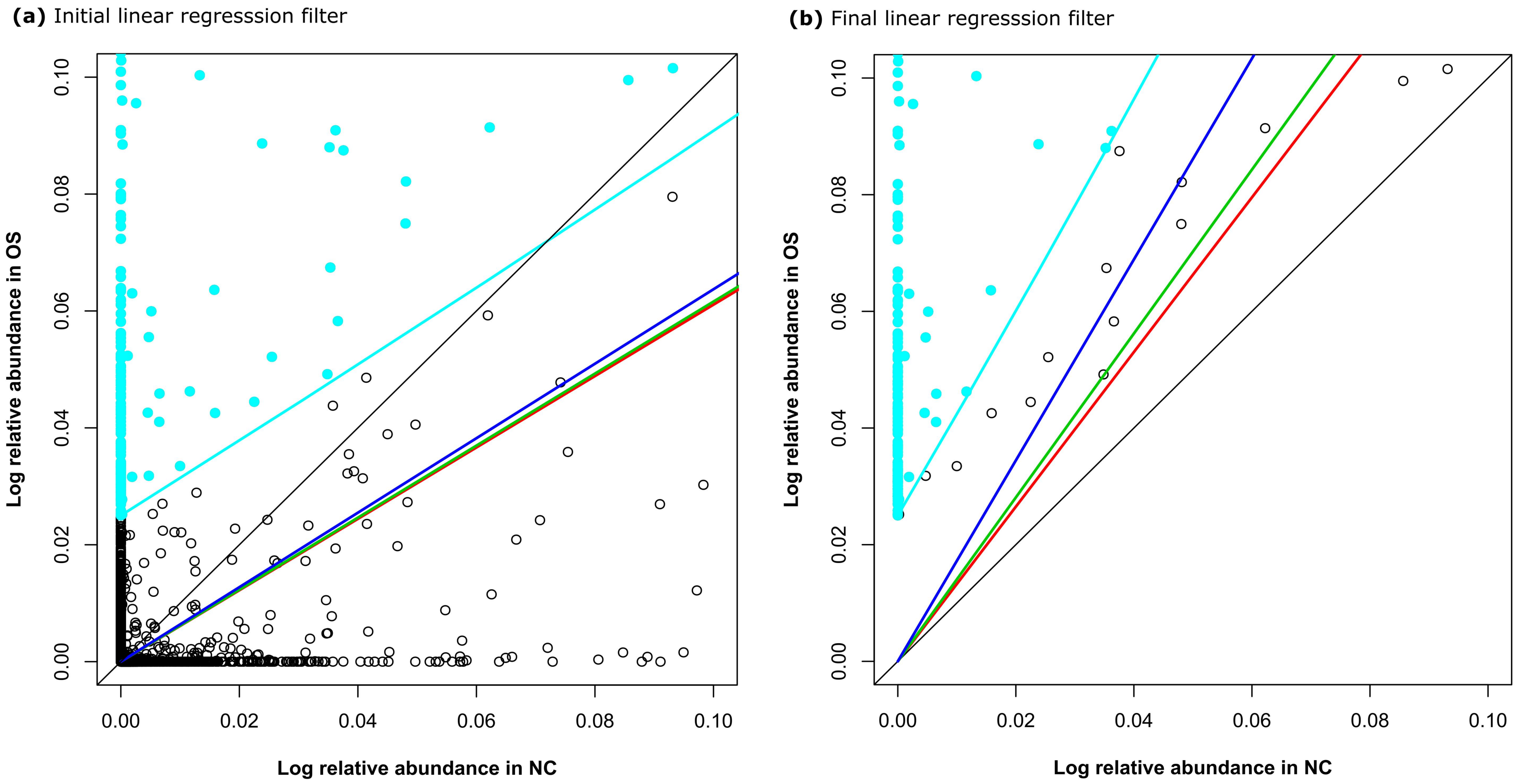

Supplementary Figure 9: Linear regression filter to determine OTUs present in ocular surface samples and retained for downstream statistical analysis (OS = ocular samples, NFW = nuclease free water; Black = 1:1 line, Red = regression line, Green = standard error line, Blue = standard error by a factor of five, Aqua = exponent of 5x error bar; Black circles = OTUs, Aqua points = final retained OS OTUs)

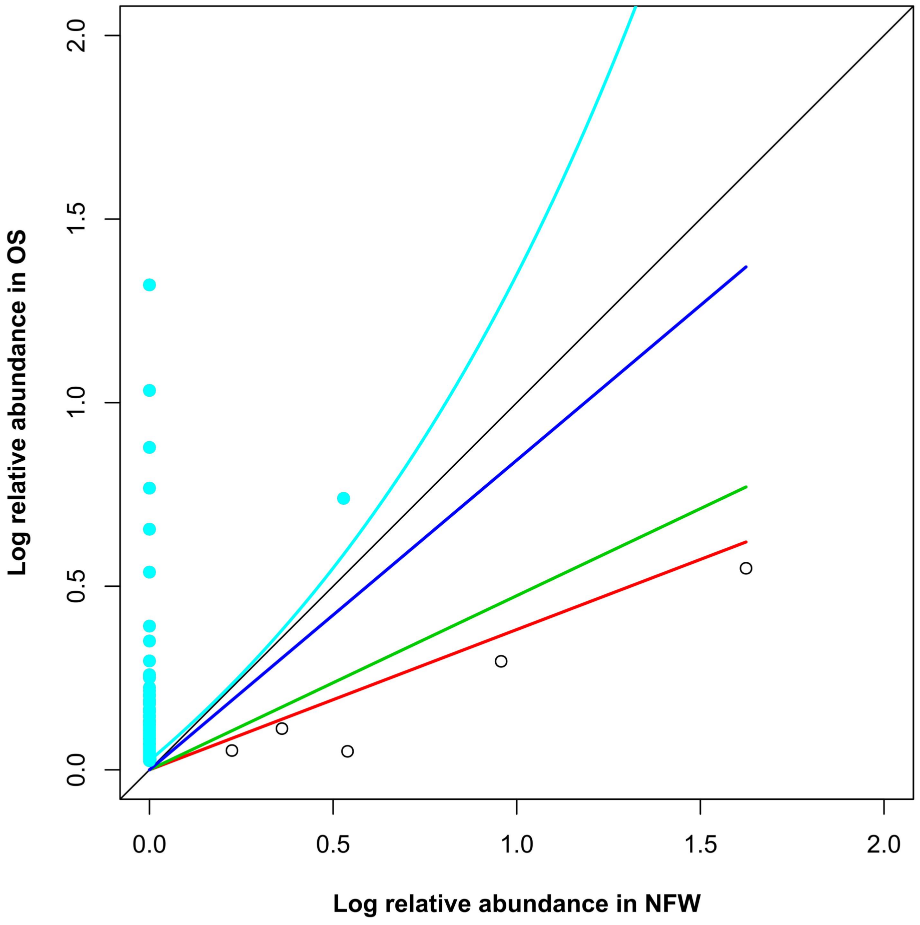

Supplement: Supplementary file 1 — Supplementary Information [file 41598_2017_10494_MOESM1_ESM.pdf]
